# Alterations in Dendritic Spine Maturation and Neurite Development Mediated by FAM19A1

**DOI:** 10.3390/cells10081868

**Published:** 2021-07-23

**Authors:** Hyo-Jeong Yong, Jong-Ik Hwang, Jae-Young Seong

**Affiliations:** 1 The GPCR Laboratory, Graduate School of Biomedical Science, Korea University College of Medicine, Seoul 02841, Korea; hjibio@korea.ac.kr; 2 Division of Research, Neuracle Science Co., Ltd., Seoul 02841, Korea

**Keywords:** FAM19A1, dendritic spines, neurite outgrowth

## Abstract

Neurogenesis and functional brain activity require complex associations of inherently programmed secretory elements that are regulated precisely and temporally. Family with sequence similarity 19 A1 (FAM19A1) is a secreted protein primarily expressed in subsets of terminally differentiated neuronal precursor cells and fully mature neurons in specific brain substructures. Several recent studies have demonstrated the importance of FAM19A1 in brain physiology; however, additional information is needed to support its role in neuronal maturation and function. In this study, dendritic spine morphology in *Fam19a1*-ablated mice and neurite development during in vitro neurogenesis were examined to understand the putative role of FAM19A1 in neural integrity. Adult *Fam19a1*-deficient mice showed low dendritic spine density and maturity with reduced dendrite complexity compared to wild-type (WT) littermates. To further explore the effect of FAM19A1 on neuronal maturation, the neurite outgrowth pattern in primary neurons was analyzed in vitro with and without FAM19A1. In response to FAM19A1, WT primary neurons showed reduced neurite complexity, whereas *Fam19a1*-decifient primary neurons exhibited increased neurite arborization, which was reversed by supplementation with recombinant FAM19A1. Together, these findings suggest that FAM19A1 participates in dendritic spine development and neurite arborization.

## 1. Introduction

Neurons are the ultimate operators of the central nervous system (CNS) and are designed to execute various CNS-specific functions. To maintain their functional integrity, each step of neuronal development and functional activity is well-regulated. During neurogenesis, postmitotic neuronal cells sprout neurites, and then axons and dendrites are specified [1,2]. Dendritic spines are postsynaptic structures that act as functional units of neurons for neural communication [3]. In conjunction with dendrite development, spinogenesis is initiated to shape neuronal circuits by connecting neurons to each other [4,5,6]. After extensive spinogenesis, neurons undergo a final refinement procedure called pruning in which unnecessary spines are eliminated in a highly coordinated manner to maintain functional efficiency [7,8,9,10]. Mature neurons are then ready to carry out neural activities via dynamic synaptic plasticity. These processes are precisely controlled by numerous extrinsic factors including secretory molecules, and it is important to evaluate their physiological mechanisms to understand neurological disorders such as neurodevelopmental disorders, dementia, and acute traumatic CNS injuries.

Among the various factors that shape neuronal integrity, secretory molecules including neuropeptides, cytokines, chemokines, and neurokines, play crucial roles as physiological signaling mediators [11,12,13,14,15]. In neurogenesis, several secretory factors are known to modulate the neuronal maturation process. For instance, oxytocin, a hypothalamic neuropeptide, stimulates voltage-gated calcium channel-dependent neurite outgrowth by regulating the gene expression of postsynaptic scaffolding proteins involved in calcium channel clustering [16]. Another hypothalamic neuropeptide, orexin-A, inhibits neurite retraction via the phospholipase D and protein kinase Cε-dependent pathway [17]. Moreover, treatment of cultured hippocampal neurons with interleukin-2 progressively enhances dendrite development and spinogenesis, especially during early neuronal maturation [5,6]. As a mediator of neuron-microglia communication, C-X3-C motif chemokine ligand 1 (CX3CL1) plays a central role in the spine pruning process. In the CNS, CX3CL1 is primarily produced by neurons and its receptor, C-X3-C motif chemokine receptor 1 (CX3CR1), is almost exclusively expressed by microglia [18,19]. It has been demonstrated that microglia lacking CX3CR1 fail to properly eliminate redundant spines, resulting in delayed spine pruning [20,21]. Furthermore, secretory molecules modulate neural signal transmission by manipulating synaptic strength. Neurotrophin-3, a secretory neurotrophic factor regulates excitatory synapse activity via organizing neurotrophin receptor tyrosine kinase C and presynaptic protein tyrosine phosphatase σ complex, which allows activation of distinct intracellular signaling cascades for synapse development [22]. Despite extensive research on CNS-specific physiological activities mediated by secretory molecules, the roles of most secretory molecules are yet to be elucidated.

Family with sequence similarity 19 A1 (FAM19A1), also referred to as TAFA1, is a secreted protein predominantly expressed in various CNS regions. Like other members of FAM19A, namely FAM19A2–FAM19A5, the mature peptide sequence of FAM19A1 is well conserved across vertebrates, suggesting that it may have evolutionarily conserved physiological functions [23]. FAM19A1 expression begins in postmitotic neurons during the early embryonic days and continues in mature excitatory neurons located in specific brain regions including limbic areas [24]. In *Fam19a1* knock-out (KO) studies, behaviors of FAM19A1-ablated mice were characterized with hyperactive locomotor activity, long-term memory deficits, fear acquisition failure and feeding behavior abnormalities [24,25]. Moreover, recent studies have shown that FAM19A1 is an active participant in several neurophysiological functions and stimulates its binding partners, G protein-coupled receptor 1 (GPR1) and neurexins (NRXNs) [26,27]. It has been suggested that FAM19A1 could act as a pan-NRXN ligand providing functional diversification to NRXNs, presynaptic membrane-bound proteins that modulate synaptic activities [27]. Some behavioral abnormalities shown in *Fam19a1* KO mice are occasionally accompanied by unusual synaptic activity [28]. Thus, the FAM19A1-ablated condition in the brain may negatively impact synaptic integrity. Although evidence indicates that FAM19A1 is a potential regulator of neural integrity, supportive studies are still needed to demonstrate the effect of FAM19A1 on neuronal maturation and function.

In this study, alterations in dendritic spine morphology in *Fam19a1* KO mice were investigated to understand spine integrity in the FAM19A1-ablated condition. The effect of FAM19A1 on neurite outgrowth progression was also examined in maturing primary neurons to identify the role of FAM19A1 in neuronal development. We found that in adult *Fam19a1* KO mice, the density and maturity of dendritic spines were lower than in adult wild-type (WT) mice with reduced dendritic arborization, indicating impairment in neuronal integrity. To investigate the effect of FAM19A1 on neuronal maturation, the primary hippocampal neuronal culture system was employed. We observed that FAM19A1 was expressed gradually across the days in vitro (DIV) and secondary neurite outgrowth was enhanced in the absence of FAM19A1. Thus, our findings show the potential roles of FAM19A1 in maintaining the physiological integrity of dendritic spines and neurite development during neurogenesis.

## 2. Materials and Methods

### 2.1. Animals and Handling

All mice were housed in a temperature-controlled (22–23 °C) facility under a 12-h light and 12-h dark photoperiod (lights on at 8:00 a.m.) with standard mouse chow and water available ad libitum. All animal procedures were approved by the Institutional Animal Care and Use Committee of Korea University (KOREA-2017-0170-C1).

The *Fam19a1 LacZ* knock-in (KI) strain was maintained by mating heterozygous *Fam19a1 LacZ* KI male mice with WT C57BL/6J female mice (The Jackson Laboratory, Bar Harbor, ME, United States). To obtain homozygous *Fam19a1 LacZ* KI mice, heterozygous *Fam19a1 LacZ* KI male mice were mated with heterozygous *Fam19a1 LacZ* KI female mice. Genotyping was performed using following primers; FWT dn1: 5′ TCG CAC AAG CAC TTA TCC AC 3′, FKI dn2: 5′ ATC TGA GTT GCT GGC TTG GT 3′ and F UP1: 5′ AGC TTC TGG GAA AGG TCT TCA 3′.

For the *Fam19a1* KO primary neuronal culture, embryos (embryonic day 17) were obtained from heterozygous *Fam19a1 LacZ* KI female mice that were mated with heterozygous *Fam19a1 LacZ* KI male mice. For the WT primary neuronal culture, pregnant C57BL/6N female mice (embryonic day 17; Koateg, Seoul, Korea) were used.

### 2.2. Dendritic Spine Analysis In Vivo

Brains were harvested from male mice on postnatal days 15, 30 and 63 (for adulthood), and stained using the FD Rapid GolgiStain kit (FD NeuroTechnologies, Columbia, MD, USA) according to the manufacturer’s instructions. The brain sections were obtained in 100-μm thickness, and the images of dendritic spines were taken using a confocal microscope (Leica, Wetzlar, Germany). The widths and lengths of the dendritic spines were measured and categorized as filopodia (lengths longer than 2 μm), long thin spines (lengths longer than 1 μm), thin spines (length–width ratios larger than 1), stubby spines (length–width ratios smaller or equal to 1), mushroom spines (widths longer than 0.6 μm), or branched spines (spines with split heads), as described previously [29]. For each experimental group, three mice were used and from each mouse, at least seven neurons were analyzed.

### 2.3. Immunohistochemical Analysis of Excitatory Synapses

Mice were perfused with phosphate-buffered saline (PBS). The brains were cross-sectioned into 50-μm sections using a vibrating microtome (Leica) and the brain sections were post fixed with 4% paraformaldehyde (PFA) in PSB. Antigen retrieval process was performed with Proteinase K (Catalog number; P2308, Sigma-Aldrich, St. Louis, MO, USA). The sections were blocked with 3% bovine serum albumin (BSA) and 0.1% Triton X-100 in PBS for 30 min at room temperature and incubated with anti-PSD95 (Catalog number; 51-6900, Invitrogen, Waltham, MA, USA) and anti-vGlut1 (Catalog number; ab5905, Millipore, Burlington, MA, USA) overnight at 4 °C. Then, the sections were incubated with the appropriate fluorescent-conjugated secondary antibodies and Hoechst 33342 (Invitrogen) for 30 min at room temperature. Images were obtained using a confocal microscope (Leica). Synaptic protein puncta were analyzed using Synapse Counter plugin from ImageJ (NIH, Bethesda, MD, USA). For each mouse, three brain sections with a section periodicity of six were analyzed and the obtained data were averaged. For each experimental group, three mice were used.

### 2.4. Dendritic Arborization Analysis In Vivo

To analyze neuronal morphology in vivo, *Fam19a1 LacZ* KI mice were bred with B6.Cg-Tg(Thy1-YFP)HJrs/J transgenic mice (The Jackson Laboratory) which express yellow fluorescent proteins in pyramidal neurons of cortical layer 5 (L5) and hippocampal CA1 and CA3. Mice were perfused with 4% PFA in PBS, and the isolated brains were post-fixed overnight. The brains were cross-sectioned into 200-μm sections using a vibrating microtome (Leica). The sections were blocked with 3% BSA and 0.5% Triton X-100 in PBS for 30 min at room temperature and incubated with anti-GFP (Catalog number; ab13970, Abcam, Cambridge, UK) overnight at 4 °C. Then, the sections were incubated with the appropriate fluorescent-conjugated secondary antibody and Hoechst 33342 (Invitrogen) for 30 min at room temperature. Images were obtained using a confocal microscope (Leica). Neurons were reconstructed using the Simple Neurite Tracer (SNT) plugin from ImageJ (NIH). Sholl analysis was performed with 10-μm radius interval using SNT plugin from ImageJ (NIH) [30]. For each experimental group, three mice were used and from each mouse, five neurons were analyzed.

### 2.5. Primary Neuronal Culture

Primary hippocampal neurons were prepared from embryos (embryonic day 17) as previously described [31]. Briefly, hippocampi were dissected in Hank’s buffered salt solution (HBSS) and digested with 2.5% trypsin for 15 min at 37 °C. The supernatant was removed, and the tissues were washed with HBSS. The tissues were gently triturated, and then dissociated cells were plated in Neurobasal^TM^ Medium (Invitrogen) supplemented with GlutaMAX (Catalog number; 25030081, Invitrogen) and 2% B-27^TM^ Supplement (Invitrogen). For Figure 4 and Figure 5, Neurobasal^TM^ Medium (Catalog number; 12348017, Invitrogen) and B-27^TM^ Supplement (Catalog number; 17504044, Invitrogen) were used. For Figure 7 and Figure S6, Neurobasal^TM^ Plus Medium (Catalog number; A3582901, Invitrogen) and B-27^TM^ Plus Supplement (Catalog number; A3582801, Invitrogen) were used. For biochemical analysis, 88 cells per mm^2^ were plated, and for imaging analysis, 65 cells per mm^2^ were plated. After 1–2 days from plating, cytosine arabinoside (AraC; 1-b-d-arabinofuranosylcytosine) was added to the culture at final concentration of 1 μM. Every 3–4 days, half of the medium from the culture dish was replaced with fresh medium.

### 2.6. RNA Isolation and Quantitative PCR

Total RNAs were isolated from primary neurons by the single-step acid guanidinium thiocyanate-phenol-chloroform method [32]. Each RNA sample was reverse-transcribed with M-MLV reverse transcriptase (Promega, Madison, WI, USA). Then, cDNAs were subjected to real-time PCR analysis with iQTM Sybr^®^ Green Supermix (Bio-Rad, Hercules, CA, USA) and the primers; *Fam19a1*_F: 5′ ATA AGT GCT TGT GCG ATG C 3′ and *Fam19a1*_R: 5′ CTC GAT GCG GTT CTT GTT AC 3′. The annealing temperature was 58 °C, and fold-changes were obtained using the 2-ΔΔCT method [33].

### 2.7. Recombinant His-Tagged FAM19A1 Protein Generation and Purification

To purify FAM19A1 protein tagged with hexahistidine at the C-terminus, the recombinant FAM19A1 protein expression plasmid was transfected into Expi293F cells (Invitrogen). After 4–5 days, the culture medium was harvested and the recombinant FAM19A1 protein was purified from the culture medium by affinity chromatography with Ni-NTA (Qiagen, Hilden, Germany) according to the manufacturer’s description.

### 2.8. Immunocytochemical Analysis for Neuronal Morphology

Primary neurons were fixed with 4% PFA at the appropriate DIV. The cells were blocked with 3% BSA and 0.1% Triton X-100 in PBS for 30 min at room temperature. Then, cells were incubated with the primary antibody, anti-Tuj1 (Catalog number; T2200, Sigma-Aldrich) for 3 h and then with the appropriate fluorescent conjugated secondary antibody and Hoechst 33342 (Invitrogen) for 30 min at room temperature. Images were obtained using a confocal microscope (Leica) and neuronal dendrites were analyzed using the SNT plugin from ImageJ (NIH). All experiments were performed in triplicate and at least 30 neurons were analyzed for each experimental group.

### 2.9. Dendritic Spine Analysis In Vitro

Primary neurons were transfected with pmaxGFP^TM^ (Lonza, Basel, Switzerland) at DIV 8 using a calcium phosphate transfection kit (Takara, Shiga, Japan) according to the manufacturer’s instructions. On the appropriate DIV, primary neurons were fixed with 4% PFA and green fluorescence signals were visualized by confocal microscopy (Leica). The in vitro dendritic spine analysis was the same as the in vivo dendritic spine analysis. All experiments were performed in triplicate and at least 30 neurons were analyzed for each experimental group.

### 2.10. Statistical Analysis

All statistical analysis was performed using Prism 5 (GraphPad Software Inc.,San Diego, CA, USA) with the data presented as mean ± standard error of the mean (SEM). Normality of the data was assessed using the Shapiro–Wilk test. Statistical significance was evaluated using the Student’s *t* test and/or one-way analysis of variance (ANOVA) with Bonferroni post-hoc test for parametric analysis and Mann–Whitney test and/or Kruskal–Wallis test with Dunn’s post-hoc test and Bonferroni correction for non-parametric analysis. A *p*-value less than 0.05 was considered statistically significant.

## 3. Results

### 3.1. Dendritic Spine Abnormalities in Cortical Neurons of Adult Fam19a1-Deficient Mice

Secretory molecules play important roles in maintaining physiological brain functions by acting as neuronal and glial signal transducers and modulators [11]. The absence of these molecules often causes dysregulated neuronal activities and consequently, the brain fails to retain its functional integrity. FAM19A1 shows subtype-specific neuronal expression in various brain regions, including the cortical layers and limbic system, and such distinctive expression patterns suggest that FAM19A1 may play major roles in maintaining specific brain functions. Functional integrity of neurons is highly related to spine plasticity, which is dynamically and sensitively adapted neuronal activity [34]. To investigate neuronal integrity in *Fam19a1*-ablated mice compared to WT mice, morphology of dendritic spines in pyramidal neurons of cortical L5, where FAM19A1 expression occurs, were analyzed [24]. For this study, homozygous *Fam19a1 LacZ* KI mice were utilized as *Fam19a1*-ablated (*Fam19a1* −/−) mice. In the previous study, it was confirmed that *Fam19a1 LacZ* KI mice do not produce the FAM19A1 protein due to disruption of the *Fam19a1* gene by the *LacZ* sequence [24].

**Figure 1:**
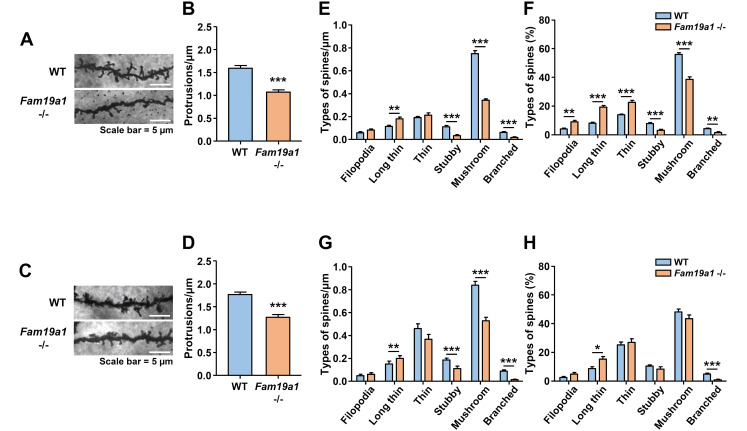
Abnormalities in dendritic spines of cortical layer 5 (L5) pyramidal neurons in adult *Fam19a1* −/− mice (postnatal day 63). (**A**) Representative images and (**B**) protrusion density of apical dendritic spines of cortical L5 neurons. (**C**) Representative images and (**D**) protrusion density of basal dendritic spines of cortical L5 neurons. (**E**) Density and (**F**) percentage of each spine type among apical dendritic spines of cortical L5 neurons. (**G**) Density and (**H**) percentage of each spine type among basal dendritic spines of cortical L5 neurons. For each experimental group, three mice were analyzed. Data are presented as means ± standard errors of means (SEM). * *p* < 0.05, ** *p* < 0.01, *** *p* < 0.001 versus WT mice by the Student’s *t* test or Mann–Whitney test with Bonferroni correction.

Pyramidal neurons in cortical L5 had fewer dendritic spines in their apical and basal dendrites in adult *Fam19a1* −/− mice than in adult WT mice (Figure 1A–D). The morphologies of dendritic spines highly correlate with the functional states of the spines [35]. In terms of dendritic spine morphology, long and thin spines are immature and branched spines are the most mature form [29]. Adult *Fam19a1* −/− mice had more long and thin spines and fewer mushroom spines than adult WT mice (Figure 1E–H). These spine alterations were also observed in hippocampal pyramidal neurons in the FAM19A1-expressing CA1 and CA3 regions of *Fam19a1* −/− mice (Figures S1 and S2) [24]. In neurons from the non-*Fam19a1*-expressing cortical layer 4 (L4), there were no significant alterations in spine density, however more immature spines were observed (Figure S3). Although there were significant alterations in dendritic spine density and maturity in cortical L5 and the hippocampus, there were no differences in number of excitatory synapses in these regions (Figure S4).

### 3.2. Fewer Mature Spines in Fam19a1 −/− Mice during Postnatal Neurodevelopment

**Figure 2:**
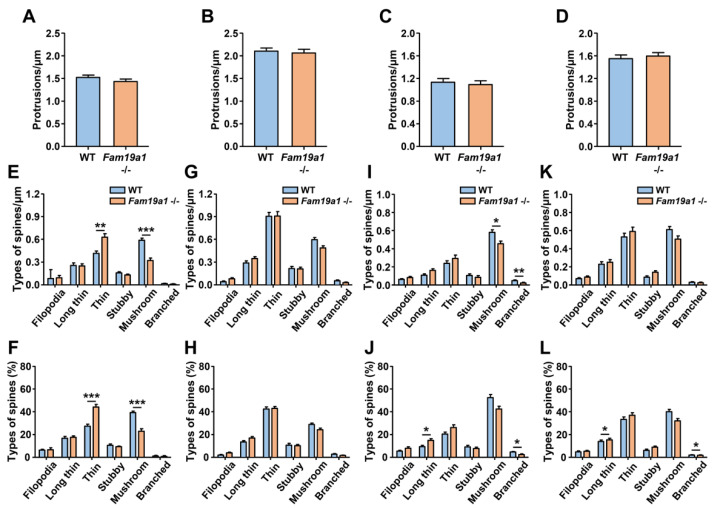
Morphological abnormalities in dendritic spines of motor cortical layer 5 (L5) pyramidal neurons in postnatal *Fam19a1* −/− mice. (**A**) Apical dendritic spine density of cortical L5 neurons at postnatal day 15 (P15). (**B**) Basal dendritic spine density of cortical L5 neurons at P15. (**C**) Apical dendritic spine density of cortical L5 neurons at postnatal day 30 (P30). (**D**) Basal dendritic spine density of cortical L5 neurons at P30. (**E**) Density and (**F**) percentage of each spine type among apical dendritic spines of cortical L5 neurons at P15. (**G**) Density and (**H**) percentage of each spine type among basal dendritic spines of cortical L5 neurons at P15. (**I**) Density and (**J**) percentage of each spine type among apical dendritic spines of cortical L5 neurons at P30. (**K**) Density and (**L**) percentage of each spine type among basal dendritic spines of cortical L5 neurons at P30. For each experimental group, three mice were analyzed. Data are presented as means ± standard errors of means (SEM). * *p* < 0.05, ** *p* < 0.01, *** *p* < 0.001 versus WT mice by Mann–Whitney test with Bonferroni correction.

The absence of FAM19A1 during early neurodevelopmental periods may lead to abnormalities in dendritic spines of adult *Fam19a1* −/− mice. Development of dendritic spines is divided into spinogenesis during early postnatal days, selective spine pruning during adolescence, and maintenance of spine dynamics during adulthood [36]. Improper execution of each developmental phase often results in malformed dendritic spine dynamics in adulthood. Thus, the morphologies of dendritic spines during spinogenesis and spine pruning were investigated to identify defects in spine formation and elimination processes. Based on previous studies, the experimental time points selected were postnatal day 15 (P15) for investigation of rapid spinogenesis and postnatal day 30 (P30) for analysis of net spine pruning in which the rate of spine pruning exceeds the rate of early spinogenesis [37,38]. Unlike the results of the dendritic spine density analysis in adult *Fam19a1* −/− mice, there were no alterations in the spine density of *Fam19a1* −/− mice at P15 and P30 (Figure 2A–D and Figure S5A,B). However, the proportions of immature spines were greater in P15 and P30 *Fam19a1* −/− mice than in P15 and P30 WT mice as observed in adult *Fam19a1* −/− mice (Figure 2E–L and Figure S5C–F). These results indicate that FAM19A1 may not participate in the initiation or elimination of early dendritic spines but may participate in the maturation and maintenance of dendritic spines. Overall, the FAM19A1 deficiency had a negative effect on dendritic spine maturation that could result in detrimental alterations in neuronal integrity.

### 3.3. Dendritic Morphology of Neurons in Fam19a1-Expressing Brain Regions

**Figure 3:**
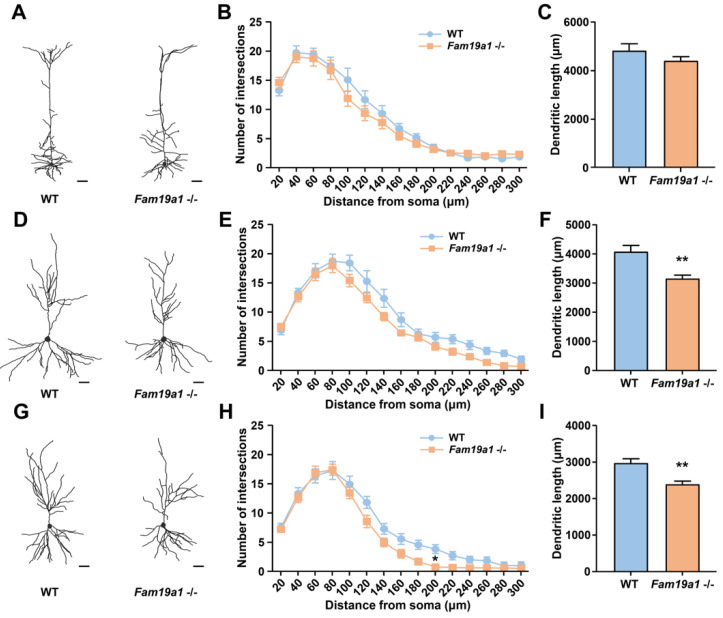
Dendritic morphology of pyramidal neurons in adult *Fam19a1* −/− mice (postnatal day 63). (**A**–**C**) Morphological analysis on pyramidal neurons of cortical layer 5 (L5). (**A**) Representative images of reconstructed cortical L5 pyramidal neurons. Scale bars represent 50 µm. (**B**) Sholl analysis and (**C**) total dendritic length of cortical L5 pyramidal neurons in wild-type (WT) and *Fam19a1* −/− mice. (**D**–**F**) Morphological analysis on pyramidal neurons of CA1 in the hippocampus. (**D**) Representative images of reconstructed CA1 pyramidal neurons. Scale bars represent 50 µm. (**E**) Sholl analysis and (**F**) total dendritic length of CA1 pyramidal neurons WT and *Fam19a1* −/− mice. (**G**–**I**) Morphological analysis on pyramidal neurons of CA3 in the hippocampus. (**G**) Representative images of reconstructed CA3 pyramidal neurons. Scale bars represent 50 µm. (**H**) Sholl analysis and (**I**) total dendritic length of CA3 pyramidal neurons in WT and *Fam19a1* −/− mice. For each experimental group, three mice were subjected for analysis. Data are presented as means ± standard errors of means (SEM). * *p* < 0.05, ** *p* < 0.01 versus WT mice by Mann–Whitney tests with Bonferroni correction.

Dendritic arborization often correlates with dendritic spine development, thus dendritic abnormality may also be present in neurons of *Fam19a1* −/− mice [39]. To investigate dendritic complexity of pyramidal neurons in *Fam19a1-*expressing brain areas, *Fam19a1 LacZ* KI mice were bred with Thy1-YFP-H transgenic mice. Pyramidal neurons in cortical L5 and hippocampal CA1 and CA3 regions were visualized, and their morphologies were examined with Sholl analysis. For pyramidal neurons of cortical L5, the number of intersections and total neurite length were not different between WT and *Fam19a1* −/− mice (Figure 3A–C). In the hippocampal CA1 regions, the number of intersections for pyramidal neurons in *Fam19a1* −/− mice was not greatly altered (Figure 3D,E), however, total neurite length was reduced in *Fam19a1* −/− mice (Figure 3F). Pyramidal neurons in hippocampal CA3 of *Fam19a1*−/− mice displayed a significant reduction in number of intersections at radius located 200 µm from the soma (Figure 3G,H), and total dendritic length was also decreased in *Fam19a1* −/− mice (Figure 3I). These data imply that pyramidal neurons located in *Fam19a1-*expressing brain regions have altered neuronal morphologies as well as impairment in dendritic spines.

### 3.4. Fam19a1 Gene Expression in Primary Neurons In Vitro

Neurogenesis requires precisely controlled expression of secretory factors at each developmental stage [12]. In an in vitro system, primary neurons undergo several stages of maturation, starting from the lamellipodia stage to the generation of dendritic spines with increasing neurite complexity [40]. During each phase of neuronal development, various secretory proteins, such as chemokines and neuropeptides, are produced to achieve neuronal maturation in vitro [13].

**Figure 4:**
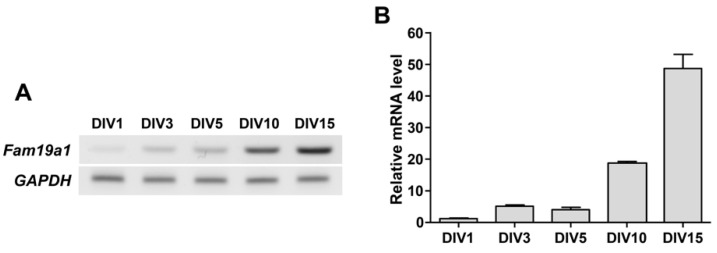
*Fam19a1* expression across the days in vitro (DIV) progression in cultured primary hippocampal neurons. (**A**) Detection of *Fam19a1* mRNA in primary hippocampal neurons. (**B**) Quantitative analysis of *Fam19a1* mRNA across the DIV progression in primary hippocampal neurons. Each experimental group was analyzed in triplicate. Data are presented as means ± standard errors of means (SEM).

To determine when the *Fam19a1* gene is expressed during the neuronal maturation process in vitro, *Fam19a1* mRNA levels in primary hippocampal neurons were examined at each DIV. *Fam19a1* gene expression increased gradually across the DIV progression, and was highest at DIV 15 when primary neurons were almost fully mature (Figure 4). In the hippocampus, *Fam19a1* expression was not observed during the embryonic stage, but was observed from the postnatal period to adulthood, indicating that *Fam19a1* is expressed at a more mature stage of neurodevelopment [24]. Thus, given that the *Fam19a1* gene was expressed in primary neurons during relatively late DIV, FAM19A1 may be required for neuronal maturation and maintenance of mature neuronal activities.

### 3.5. Increased Dendritic Complexity in Fam19a1-Deficient Primary Neurons

**Figure 5:**
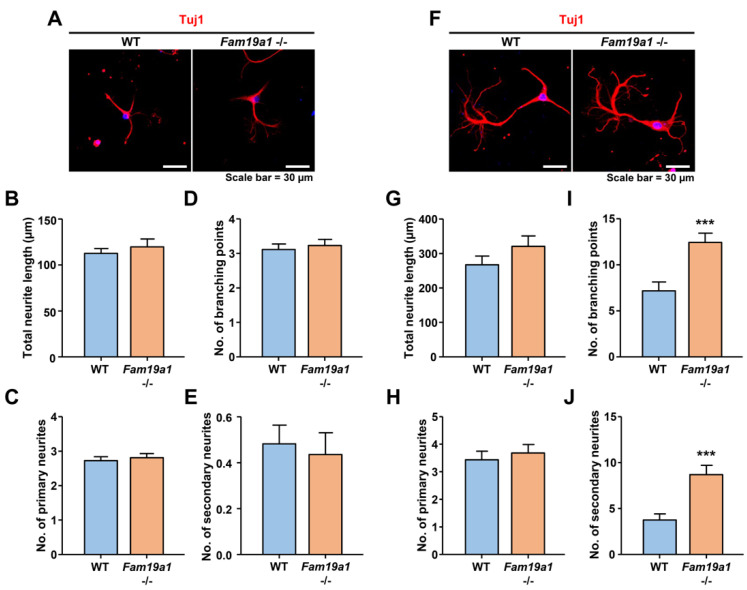
Abnormal neurite formation in *Fam19a1* −/− primary hippocampal neurons. (**A**–**E**) Primary hippocampal neurons at days in vitro 3 (DIV 3). (**A**) Representative images of WT and *Fam19a1* −/− neurons at DIV 3. Neuronal morphologies were analyzed in terms of total neurite length; (**B**), total number of primary neurites; (**C**), total number of branching points; (**D**), and total number of secondary neurites; (**E**). (**F**–**J**) Primary hippocampal neurons at days in vitro 6 (DIV 6). (**F**) Representative images of WT and *Fam19a1* −/− neurons at DIV 6. Neuronal morphologies were analyzed in terms of total neurite length; (**G**), total number of primary neurites; (**H**), total number of branching points; (**I**), and total number of secondary neurites; (**J**). Experiments were performed in triplicate and at least 30 neurons were analyzed for each experimental group. Data are presented as means ± standard errors of means (SEM). *** *p* < 0.001 versus WT by Mann–Whitney tests.

To investigate developmental progression of neurons in the absence of FAM19A1, primary hippocampal neurons from WT and *Fam19a1* −/− mice were cultured. For neuronal morphological analysis, total neurite length, number of branching points, and number of primary and secondary neurites were examined. At DIV 3, there were no morphological differences between WT and *Fam19a1* −/− primary neurons (Figure 5A–E). At DIV 6, total neurite length and number of primary neurites did not differ between *Fam19a1* −/− and WT primary neurons (Figure 5F–H), however, the numbers of branching points and secondary neurites were higher in *Fam19a1* −/− than in WT primary neurons (Figure 5I,J). These data suggest that FAM19A1 ablation altered the later stage of neurite development rather than initial neurite outgrowth.

### 3.6. Reduced Dendritic Arborization in Primary Neurons upon Treatment with Recombinant FAM19A1

**Figure 6:**
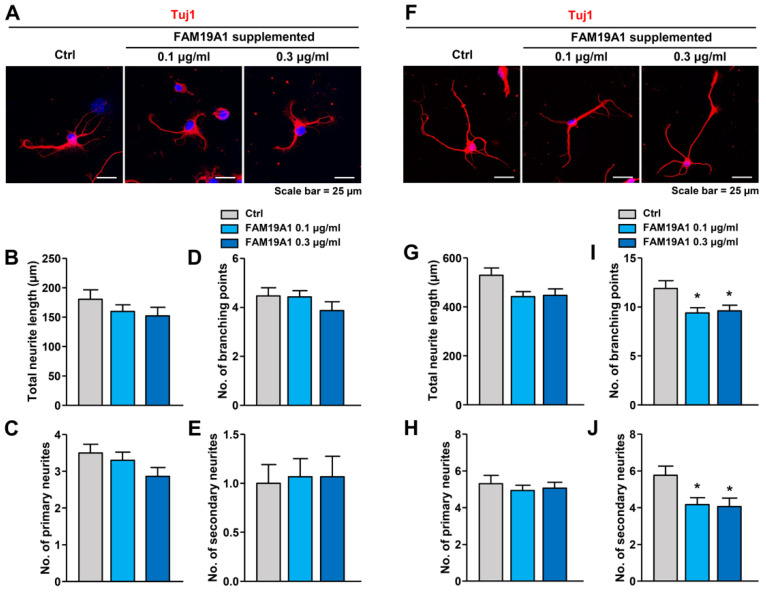
Neuronal morphology of his-tagged FAM19A1-treated wild-type (WT) primary hippocampal neurons. (**A**–**E**) Primary hippocampal neurons at days in vitro 3 (DIV 3). (**A**) Representative images of non-treated (Ctrl) and his-tagged FAM19A1-treated WT primary hippocampal neurons at DIV 3. Neuronal morphologies were analyzed in terms of total neurite length; (**B**), total number of primary neurites; (**C**), total number of branching points; (**D**), and total number of secondary neurites; (**E**). (**F**–**J**) Primary hippocampal neurons at days in vitro 6 (DIV 6). (**F**) Representative images of non-treated (Ctrl) and his-tagged FAM19A1-treated WT primary hippocampal neurons at DIV 6. Neuronal morphologies were analyzed in terms of total neurite length; (**G**), total number of primary neurites; (**H**), total number of branching points; (**I**), and total number of secondary neurites; (**J**). Experiments were performed in triplicate and at least 30 neurons were analyzed for each experimental group. Data are presented as means ± standard errors of means (SEM). * *p* < 0.05 versus non-treated by one-way analysis of variance (ANOVA) with Bonferroni post-hoc test.

To further investigate the role of FAM19A1 in neurite generation, WT primary hippocampal neurons were treated with recombinant FAM19A1, and morphological changes were analyzed. Overexpressed recombinant FAM19A1 protein tagged with hexahistidine at the C-terminus was purified from a mammalian cell culture system. At DIV 1, WT primary hippocampal neurons were treated with recombinant FAM19A1 and harvested at DIV 3. There was no difference between the non-treated control and the recombinant FAM19A1-treated groups at DIV 3 (Figure 6A–E). The primary neurons were further treated with recombinant FAM19A1 at DIV 4 and harvested at DIV 6. At DIV 6, recombinant FAM19A1-treated primary neurons showed no alteration in the total neurite length and the number of primary neurites (Figure 6F–H), but the numbers of branching points and secondary neurites were lower in recombinant FAM19A1-treated than in non-treated primary neurons (Figure 6I,J). Thus, the observed reduction in the number of branching points in the treated group is primarily due to reduction in the number of secondary neurites.

### 3.7. Increase in Neurite Complexity in Fam19a1-Deficient Primary Neurons Was Reversed by Recombinant FAM19A1 Supplementation

**Figure 7:**
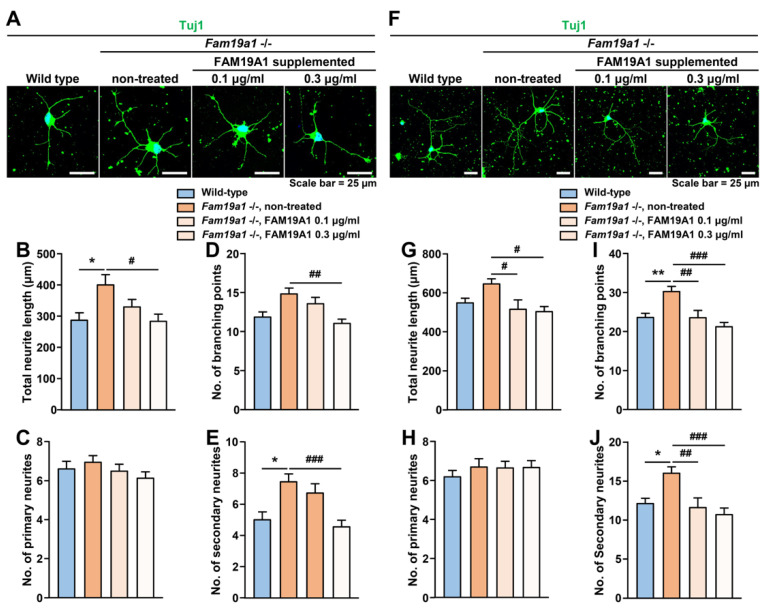
Morphological alterations in neurites of *Fam19a1* −/− primary hippocampal neurons was reversed by His-tagged FAM19A1 supplementation. (**A**–**E**) Primary hippocampal neurons at days in vitro 3 (DIV 3). (**A**) Representative images of wild-type (WT) and *Fam19a1* −/− neurons with or without his-tagged FAM19A1 supplementation. Neuronal morphologies were analyzed in terms of total neurite length; (**B**), total number of primary neurites; (**C**), total number of branching points; (**D**), and total number of secondary neurites; (**E**). (**F**–**J**) Primary hippocampal neurons at days in vitro 6 (DIV 6). (**F**) Representative images of WT and *Fam19a1* −/− neurons with or without his-tagged FAM19A1 supplementation. Neuronal morphologies were analyzed in terms of total neurite length; (**G**), total number of primary neurites; (**H**), total number of branching points; (**I**), and total number of secondary neurites; (**J**). Experiments were performed in triplicate and at least 30 neurons were analyzed for each experimental group. Data are presented as means ± standard errors of means (SEM). * *p* < 0.05, ** *p* < 0.01 versus WT and # *p* < 0.05, ## *p* < 0.01, ### *p* < 0.001 versus non-treated *Fam19a1* −/− by one-way analysis of variance (ANOVA) with the Bonferroni post-hoc test or Kruskal–Wallis test with Dunn’s post-hoc test.

To determine whether the observed abnormal neurite arborization in *Fam19a1*-deficient primary neurons was due to the absence of FAM19A1, *Fam19a1*-deficient primary neurons were supplemented with recombinant FAM19A1, and neurite formation was examined. *Fam19a1*-deficient primary neurons were treated with recombinant FAM19A1 at DIV 1 and 4 and harvested at DIV 3 and 6. There was an increase in neurite complexity in *Fam19a1*-deficient primary neurons, compared to the non-treated WT primary neurons at DIV 3 (Figure 7A–E), and such abnormalities were reversed upon recombinant FAM19A1 supplementation. A similar trend was also observed at DIV 6 (Figure 7F–J). These data indicates that the neurite abnormality in *Fam19a1*-deficient primary neurons is due to the absence of FAM19A1.

In brief, the absence of FAM19A1 in the culture condition increases neurite arborization, and this alteration can be reversed by FAM19A1 supplementation. Moreover, excessive FAM19A1 supplementation to WT primary neuronal culture reduced neurite complexity. The *Fam19a1* expression pattern in primary neurons across the DIV progression and the morphologies of primary neurons with and without FAM19A1 suggest that FAM19A1 modulates later neuronal development progression.

### 3.8. Morphological Analysis of Dendritic Spines in Primary Neurons Supplemented with FAM19A1

Because abnormal dendritic arborization often results in impaired dendritic spine development [39], the morphology of dendritic spines in *Fam19a1*-deficient primary neurons may also be altered. Unfortunately, we could not investigate dendritic spine morphology in *Fam19a1*-deficient primary neurons because we often failed to maintain *Fam19a1*-deficient primary neurons longer than DIV 7–8 due to unidentified reasons.

To investigate the effect of excessive FAM19A1 supplementation on dendritic spines in primary neurons, WT primary hippocampal neurons were treated with recombinant FAM19A1 at DIV 1, 4, 7, 10, and 13 and harvested at DIV 10 and 15. To visualize dendritic spines, primary neurons were transfected with a plasmid expressing green fluorescence protein at DIV 8. At DIV 10, there was no difference in dendritic spine density between the experimental groups, but primary neurons supplemented with FAM19A1 showed reduced maturity compared to non-treated primary neurons (Figure S6A–D). However, at DIV 15, there were no notable alterations in spine density or morphology in the FAM19A1-treated groups (Figure S6E–H). Based on the *Fam19a1* expression pattern across the DIV progression shown in Figure 4, a substantial amount of endogenous FAM19A1 derived from primary neurons may have accumulated in the culture condition by DIV 15, thus excessive FAM19A1 supplementation may not have caused differences in dendritic spine morphology.

## 4. Discussion

FAM19A1 is a secreted protein expressed by specific subset of neurons located within several brain areas [24]. It has been demonstrated that FAM19A1 mediates post-translational modification of neurexins, a family of presynaptic adhesion molecules involved in synaptic function [27]. In addition, ablation of FAM19A1 in mice led to several behavior abnormalities related to aberrant synaptic activities [24,25]. Based on these findings, it has been suggested that FAM19A1 maintains neural activities in a neuronal cell type-specific manner. Moreover, FAM19A1 expression in post-differentiated neuronal cells during early embryonic neurodevelopment suggests it may play a role in neuronal maturation [24]. In this study, alterations in the structural plasticity of dendritic spines in *Fam19a1*-ablated mice and the effect of FAM19A1 on neurite development during in vitro neurogenesis were investigated to determine the role of FAM19A1 in neural integrity.

Based on the expression profile of *Fam19a1* across in vivo neurodevelopmental stages, *Fam19a1* expression was observed in more mature than premature neurons, suggesting an association between *Fam19a1* expression and neuronal maturity [24]. A comparable expression pattern was observed during in vitro development of primary neurons, as *Fam19a1* expression increased gradually across the DIV. In in vitro neurogenesis, premature neurons undergo developmental progression similar to that in vivo [2]. To obtain complex dendritic arborization, premature neurons in the lamellipodia state initially attempt to branch out neurites, and upon generation of primary neurites, development of secondary neurites commences [41]. Interestingly, in culture conditions with or without FAM19A1, primary neurite development was unaffected, but secondary neurite outgrowth was significantly altered. The alterations in secondary neurite development, but not in primary neurites, suggests that FAM19A1 does not play a role in initial neurite generation, but rather participates in later stage of neuronal development and maturation.

Dendritic spines are protrusive membrane structures that serve as postsynaptic sites in synapses and that primarily receive excitatory inputs [42]. The physiological states of dendritic spines are tremendously dynamic, depending on the neuronal activities and morphologies of dendritic spines correlate with their maturity, which occasionally reflects synaptic strength [3,43]. Improper maintenance of dendritic spine density and maturity often leads to imbalanced excitatory and inhibitory synaptic inputs causing failures in neural signal transmission [44]. There have been several studies that demonstrated correlations between dendritic spine integrity and abnormal behaviors, for instance, an overexpression transgenic mouse model of *Shank3*, SH3 and multiple ankyrin repeat domains 3 showed manic-like behavior with increased dendritic spine density [45]. In addition, *Jmjd2b*, jumonji domain containing 2B-deficient mice displayed hyperactive behavior and memory deficits with increased spine density but decreased synaptic maturity [46,47]. Observed dendritic spine conditions and previously reported abnormal behaviors in *Fam19a1* −/− mice may imply impaired synaptic activities in *Fam19a1* −/− mice. This hypothesis could be further supported by potential functional diversification of NRXNs mediated by FAM19A1 [27]. Neuroligins (NLGNs) and NRXNs are synaptic organizing membrane-bound molecules that form trans-synaptic bridges to promote synapse development [48]. Recent studies have demonstrated that the MAM domain-containing glycosylphosphatidylinositol anchor regulates trans-synaptic bridge formation by binding to the same site on NLGNs that NRXNs bind to [49]. Similarly, because it is a ligand for pan-neurexin, FAM19A1 may alter the binding between NRXNs and NLGNs, leading to diversification of physiological synaptic states and changes in synaptic transmissions.

Like dendritic spines, dendrites are also dynamically modulated over time and their complexity highly correlates with spine maturity [9,50]. Malformation of dendritic arborization often cause impairment in dendritic spine development. On the other hand, stabilization of dendritic spines contributes to maintaining dendritic architecture over time as reduced integrity of synapses leads to structural simplification of dendrites [51]. Although neurogenesis is a sequential event in which neurite outgrowth commences before spinogenesis, the integrity of dendritic spines can shape development of the neurite branching pattern [41]. Postsynaptic density protein 95 (PSD95) has been shown to strongly correlate with synaptic stability by contributing to spine maturation. In a recent study, PSD95 knockdown in neurons led to an increase in dendrite complexity [52]. This suggests that neurite and dendritic spine development do not follow a simple serial progression, but rather overlap and affect each other during neurogenesis. The dendritic spines in *Fam19a1* −/− mice were less mature than those in WT mice with reduced dendrite arborization, however, the neurites of *Fam19a1* −/− primary neurons were more mature than that of WT primary neurons showing acceleration of neuronal maturation. Given that FAM19A1 is a potential regulator for synapse organization, FAM19A1 may play important roles in maintaining synaptic integrity, but may also serve as a negative regulator of neurite arborization during neurodevelopment.

In summary, this study demonstrates the effect of FAM19A1 on neuronal cells in vivo and in vitro. In adult *Fam19a1* −/− mice, neurons in the *Fam19a1*-expressing cortical layer and hippocampal regions showed low dendritic spine density with immature spine morphologies. Dendritic spine analysis on P15 and P30 suggests that the impairment observed in the adult stage is not related to early developmental spinogenesis or spine pruning, but rather is a result of failure in dendritic spine maintenance. To further investigate the role of FAM19A1 in neurodevelopment, the in vitro primary neuronal culture system was employed. *Fam19a1* gene expression in primary neurons increased in a DIV-dependent manner, and the presence of FAM19A1 in the primary neuronal culture modulated secondary neurite complexity. This study provides preliminary evidence showing that FAM19A1 contributes to neural integrity by regulating dendritic spine maturation and neurite complexity.

## Data Availability

The data presented in this study are available on request from the GPCR laboratory of Korea University, and there are no restrictions on data availability.

## Supplementary Materials

The following are available online at https://www.mdpi.com/article/10.3390/cells10081868/s1, Figure S1: Dendritic spine morphology of hippocampal CA1 pyramidal neurons in adult *Fam19a1* −/− mice (postnatal day 63), Figure S2: Dendritic spine morphology of hippocampal CA3 pyramidal neurons in adult Fam19a1 −/− mice (postnatal day 63), Figure S3: Dendritic spine morphology of motor cortical layer 4 spiny stellate neurons in adult Fam19a1 −/− mice (postnatal day 63), Figure S4: PSD95 and vGlut1 expressions in adult Fam19a1 −/− mice (postnatal day 63), Figure S5: Dendritic spine morphology of motor cortical layer 4 (L4) spiny stellate neurons in postnatal Fam19a1 −/− mice, and Figure S6: Spine morphology of his-tagged FAM19A1-treated wild-type (WT) primary hippocampal neurons. Submitted in a separate file.
