# Supplementary material for: Alterations in Dendritic Spine Maturation and Neurite Development Mediated by FAM19A1
Source: Cells. 2021 Jul 23;10(8):1868. doi: 10.3390/cells10081868 (PMC8392516; doi:10.3390/cells10081868)
Supplement: Supplementary file 1 [file cells-10-01868-s001.zip › cells-1158273-supplementary.pdf]

Alterations in dendritic spine maturation and neurite development mediated by FAM19A1

Hyo-Jeong Yong [1,*], Jong-Ik Hwang [1] and Jae-Young Seong [1,2,*]

[1] The GPCR laboratory, Graduate School of Biomedical Science, Korea University College of Medicine, Seoul, 02841, Korea
[2] Division of Research, Neuracle Science Co., Ltd., Seoul, 02841, Korea
* Correspondence: kylie0707@korea.ac.kr (H.-J.Y .); jyseong@korea.ac.kr (J.-Y.S.); Tel.: +82-2-2286-1090 (J.-Y.S.)

Supplementary Information

Supplementary figures

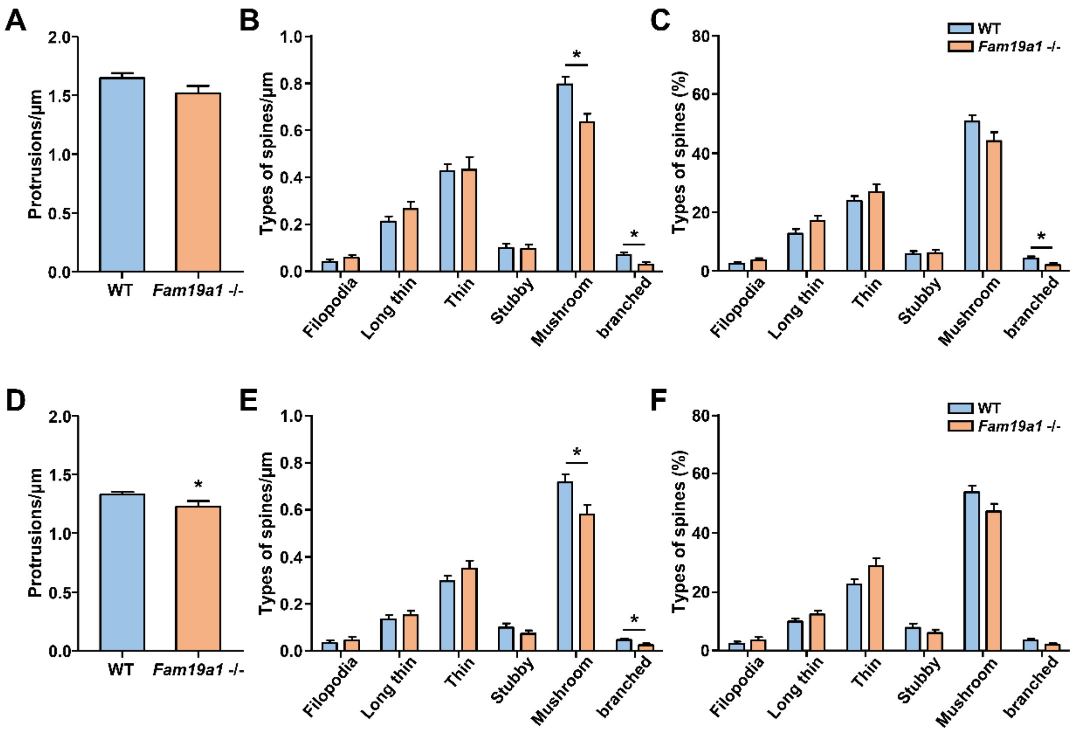

Supplementary figure S1. Dendritic spine morphology of hippocampal CA1 pyramidal neurons in adult *Fam19a1* -/- mice (postnatal day 63). (A–C) Analysis on apical dendritic spines. (A) Dendritic spine density and (B) density and (C) percentage of each spine type. (D–F) Analysis on basal dendritic spines. (D) Dendritic spine density and (E) density and (F) percentage of each spine type. For each experimental group, three mice were analyzed. Data are presented as means ± standard errors of means (SEM). *$p < 0.05$ versus WT mice by Student's *t* test or Mann-Whitney test with Bonferroni correction.

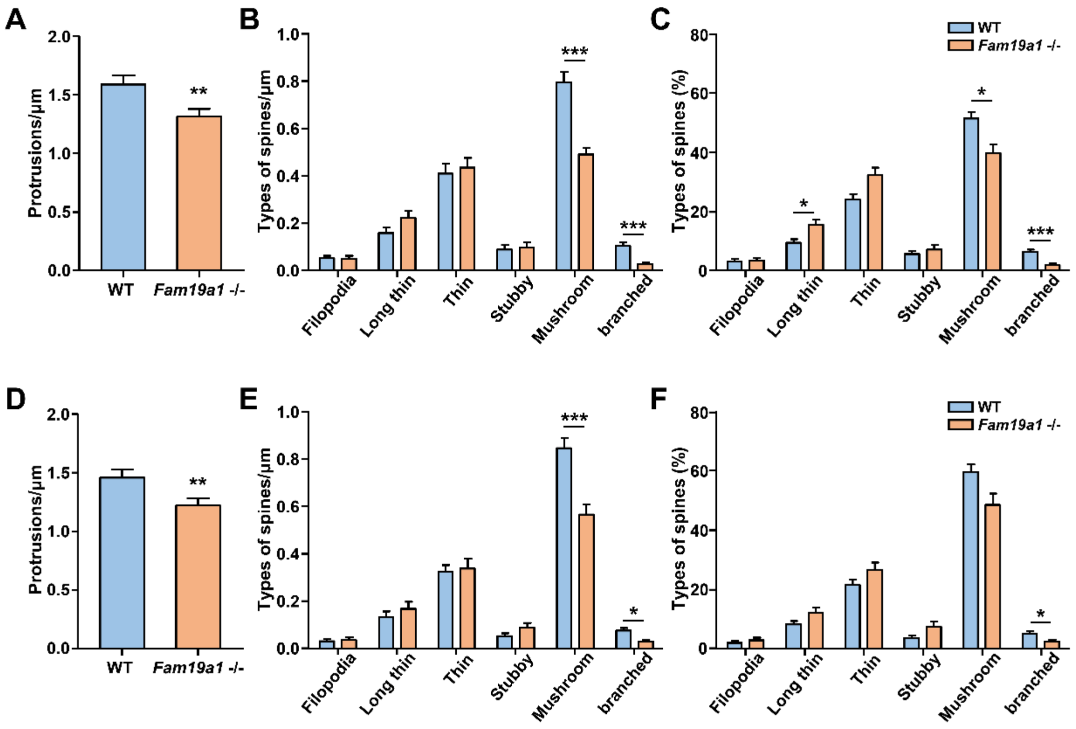

Supplementary figure S2. Dendritic spine morphology of hippocampal CA3 pyramidal neurons in adult *Fam19a1 -/-* mice (postnatal day 63). (A–C) Analysis on apical dendritic spines. (A) Dendritic spine density and (B) density and (C) percentage of each spine type. (D–F) Analysis on basal dendritic spines. (D) Dendritic spine density and (E) density and (F) percentage of each spine type. For each experimental group, three mice were analyzed. Data are presented as means ± standard errors of means (SEM). *$p < 0.05$, **$p < 0.01$, ***$p < 0.001$ versus WT mice by Student's t tests or Mann-Whitney test with Bonferroni correction.

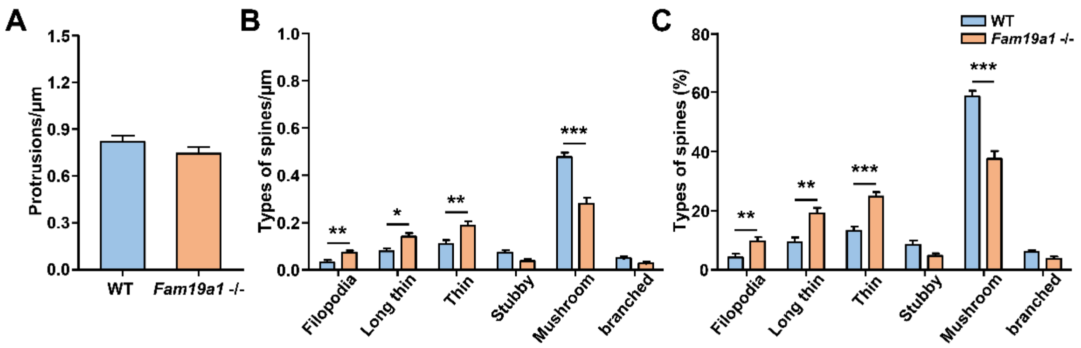

Supplementary figure S3. Dendritic spine morphology of motor cortical layer 4 spiny stellate neurons in adult *Fam19a1* -/- mice (postnatal day 63). (A) Dendritic spine density and (B) density and (C) percentage of each spine type. For each experimental group, three mice were analyzed. Data are presented as means ± standard errors of means (SEM). *$p < 0.05$, **$p < 0.01$, ***$p < 0.001$ versus WT mice by Mann-Whitney test with Bonferroni correction.

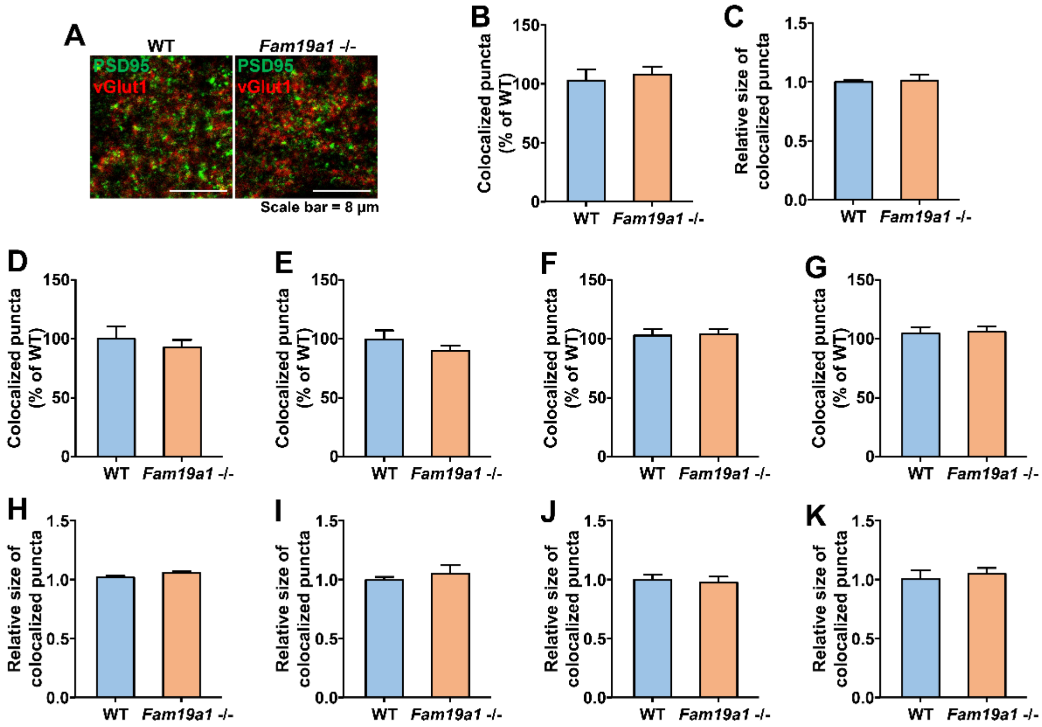

Supplementary figure S4. PSD95 and vGlut1 expressions in adult *Fam19a1* -/- mice (postnatal day 63). (A) Representative images for PSD95 (green) and vGlut1 (red) expressions in motor cortical layer 5 (L5) of wild-type (WT) and *Fam19a1* -/- mice. (B) Relative percentage of colocalized puncta (PSD95 and vGlut1) in motor cortical L5. (C) Relative size of colocalized puncta in motor cortical L5. (D–G) Relative percentage of colocalized puncta (PSD95 and vGlut1) in the hippocampus. (D); CA1 stratum oriens, (E); CA1 stratum radiatum, (F); CA3 stratum oriens, and (G); CA3 stratum radiatum. (H–K) Relative size of colocalized puncta in the hippocampus. (H); CA1 stratum oriens, (I); CA1 stratum radiatum, (J); CA3 stratum oriens, and (K); CA3 stratum radiatum. For each experimental group, three mice were analyzed. Data are presented as means ± standard errors of means (SEM).

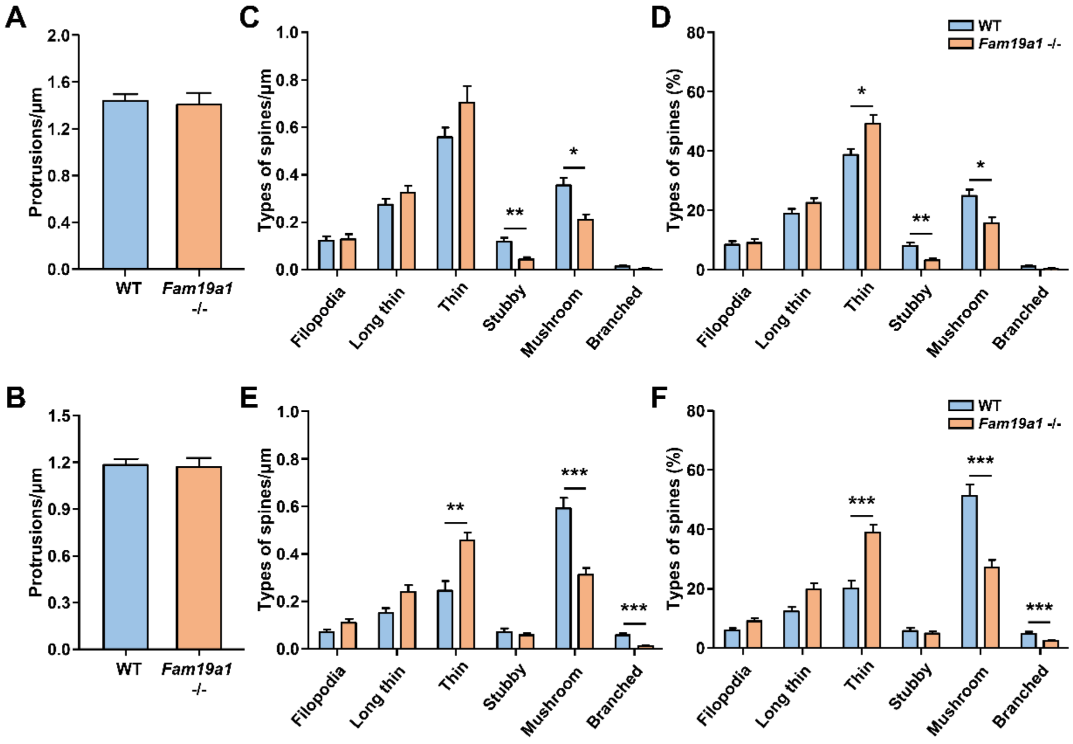

Supplementary figure S5. Dendritic spine morphology of motor cortical layer 4 (L4) spiny stellate neurons in postnatal *Fam19a1* -/- mice. (A) Dendritic spine density of cortical L4 neurons for postnatal day 15 (P15). (B) Dendritic spine density of cortical L4 neurons for postnatal day 30 (P30). (C) Density and (D) percentage of each spine type for cortical L4 neurons at P15. (E) Density and (F) percentage of each spine type for cortical L4 neurons at P30. For each experimental group, three mice were analyzed. Data are presented as means ± standard errors of means (SEM). **p* < 0.05, ***p* < 0.01, ****p* < 0.001 versus WT mice by Mann-Whitney test with Bonferroni correction.

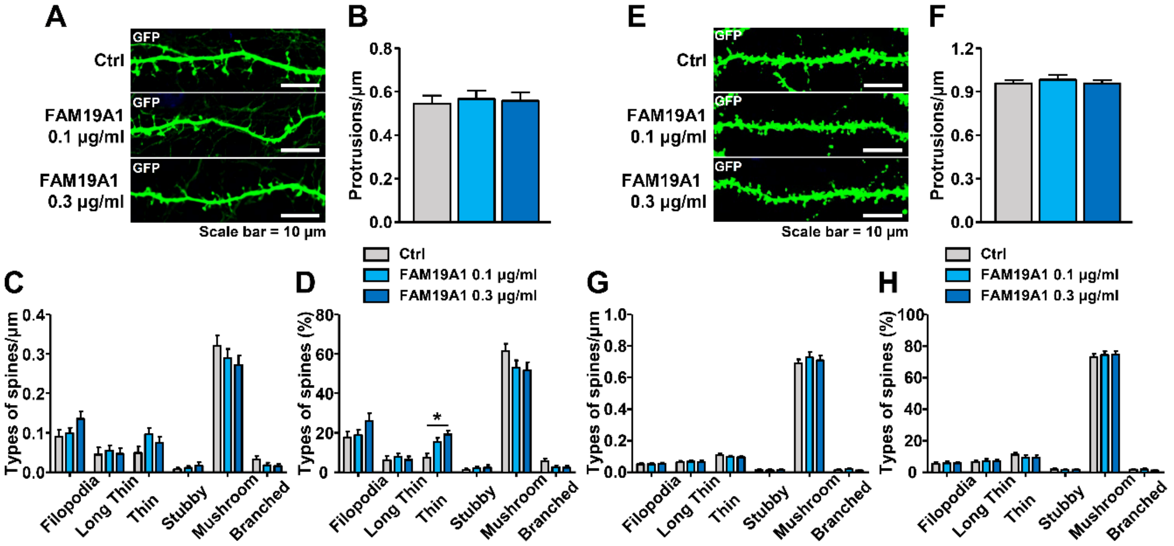

Supplementary figure S6. Spine morphology of his-tagged FAM19A1-treated wild-type (WT) primary hippocampal neurons. (A–D) Analysis of dendritic spines at days *in vitro* 10 (DIV 10). (A) Representative images of non-treated (Ctrl) and his-tagged FAM19A1-treated WT dendrites at DIV 10. (B) Dendritic spine density, (C) density and (D) percentage of each spine type. (E–H) Analysis of dendritic spines at days *in vitro* 15 (DIV 15). (A) Representative images of non-treated (Ctrl) and his-tagged FAM19A1-treated WT dendrites at DIV 15. (F) protrusion density, (G) density of each spine type and (H) percentage of each spine type. Experiments were performed in triplicate and at least 30 neurons were analyzed for each experimental group. Data are presented as means ± standard errors of means (SEM). *$p < 0.05$ versus WT mice by Kruskal-Wallis test with Dunn's post-hoc test and Bonferroni correction.
